# *catena*-Poly[[[dichlorido(pyridin-1-ium-3-yl)arsenic(III)]-μ-chlorido] mono­hydrate]

**DOI:** 10.1107/S1600536812042882

**Published:** 2012-11-03

**Authors:** Lukas Reck, Wolfgang Schmitt

**Affiliations:** a School of Chemistry, Trinity College Dublin, Dublin 2, Ireland

## Abstract

The crystal structure of the title compound, {[AsCl_3_(C_5_H_5_N)]·H_2_O}_*n*_, is characterized by polymeric chains consisting of alternating arsenic and chlorine atoms running parallel to the *a* axis. O—H⋯Cl and N—H⋯O hydrogen bonds mediated by non-coordinating water mol­ecules assemble these chains into a three-dimensional framework. The As^III^ atom, the atoms of the pyridinium ring and the water O atom have *m* site symmetry and the bridging Cl atom has site symmetry 2. This is the first reported organotrichloro­arsenate(III) in which arsenic adopts a ψ-octa­hedral fivefold coordination.

## Related literature

For the synthesis, see: Binz & von Schickh (1936 ▶). For mono­meric and oligomeric monoorganohaloarsenates(III) with tetra­coordinate arsenic, see: Grewe *et al.* (1998 ▶). For homologous monoorganohaloanti­monate(III)/-bis­muth­ate(III) structures, see: Althaus *et al.* (1999 ▶); Breunig *et al.* (1992 ▶, 1999 ▶, 2010 ▶); Hall & Sowerby (1988 ▶); James *et al.* (1999 ▶); Preut *et al.* (1987 ▶); Sheldrick & Martin (1992 ▶); von Seyerl *et al.* (1986 ▶). For organoarsenic functionalized metal oxide clusters, see: Breen, Clérac *et al.* (2012 ▶); Breen, Zhang *et al.* (2012 ▶); Onet *et al.* (2011 ▶); Zhang *et al.* (2012 ▶).
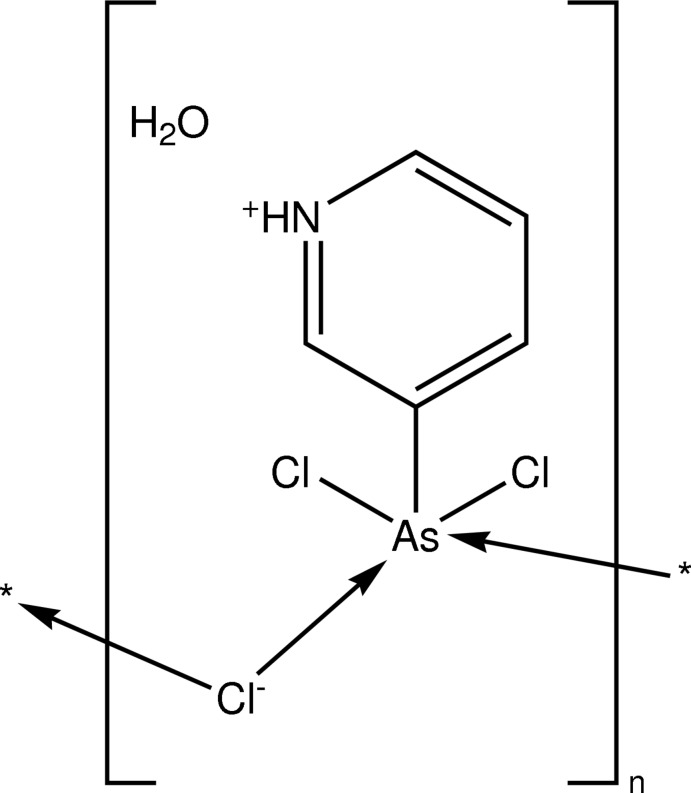


## Experimental

### 

#### Crystal data

[AsCl_3_(C_5_H_5_N)]·H_2_O
*M* *_r_* = 278.39
Orthorhombic, 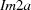

*a* = 8.2107 (9) Å
*b* = 8.5812 (9) Å
*c* = 13.2046 (14) Å
*V* = 930.37 (17) Å^3^
*Z* = 4
Mo *K*α radiation
μ = 4.46 mm^−1^
*T* = 200 K
0.5 × 0.2 × 0.2 mm

#### Data collection

Bruker SMART APEX CCD diffractometer
Absorption correction: multi-scan (*SADABS*; Bruker, 1997 ▶) *T*_min_ = 0.341, *T*_max_ = 0.469
3423 measured reflections
1184 independent reflections
1167 reflections with *I* > 2σ(*I*)
*R*_int_ = 0.068

#### Refinement

*R*[*F* ^2^ > 2σ(*F* ^2^)] = 0.032
*wR*(*F* ^2^) = 0.078
*S* = 1.10
1184 reflections
66 parameters
3 restraints
H atoms treated by a mixture of independent and constrained refinement
Δρ_max_ = 1.14 e Å^−3^
Δρ_min_ = −1.28 e Å^−3^
Absolute structure: Flack (1983 ▶), 529 Friedel pairs
Flack parameter: 0.006 (12)

### 

Data collection: *SMART* (Bruker, 1997 ▶); cell refinement: *SAINT* (Bruker, 1997 ▶); data reduction: *SAINT*; program(s) used to solve structure: *SHELXS97* (Sheldrick, 2008 ▶); program(s) used to refine structure: *SHELXL97* (Sheldrick, 2008 ▶); molecular graphics: *OLEX2* (Dolomanov *et al.*, 2009 ▶); software used to prepare material for publication: *OLEX2*.

## Supplementary Material

Crystal structure: contains datablock(s) global. DOI: 10.1107/S1600536812042882/nk2180sup1.cif

Structure factors: contains datablock(s) I. DOI: 10.1107/S1600536812042882/nk2180Isup2.hkl

Supplementary material file. DOI: 10.1107/S1600536812042882/nk2180Isup3.cdx

Additional supplementary materials:  crystallographic information; 3D view; checkCIF report

## Figures and Tables

**Table 1:** Selected bond lengths (Å)

As1—Cl1	2.2624 (8)
As1—Cl2	2.8907 (5)

**Table 2:** Hydrogen-bond geometry (Å, °)

*D*—H⋯*A*	*D*—H	H⋯*A*	*D*⋯*A*	*D*—H⋯*A*
O1—H1⋯Cl2	0.82 (2)	2.40 (2)	3.197 (2)	165 (3)
N3—H3⋯O1^i^	0.88	1.84	2.711	173

Symmetry code: (i) 
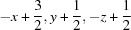
.

**Fig. 1.**
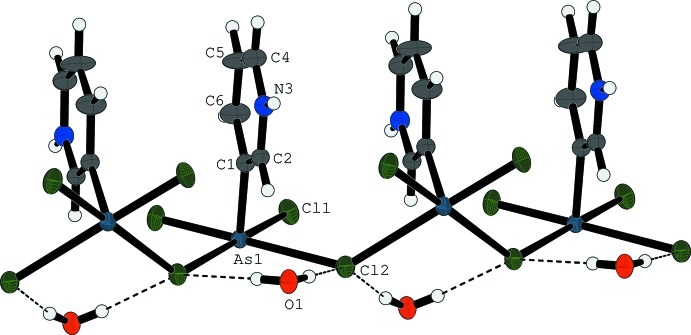
Polymeric [C5H5NAsCl3]∞ chain with O—H···Cl hydrogen bonds to neighbouring water molecules, showing the labelling scheme in the asymmetric unit. Thermal displacement ellipsoids are drawn at the 50% probability level. Hydrogen bonds are indicated by dashed lines. Colour code: H white, C grey, N blue, O red, Cl green, As teal.

**Fig. 2.**
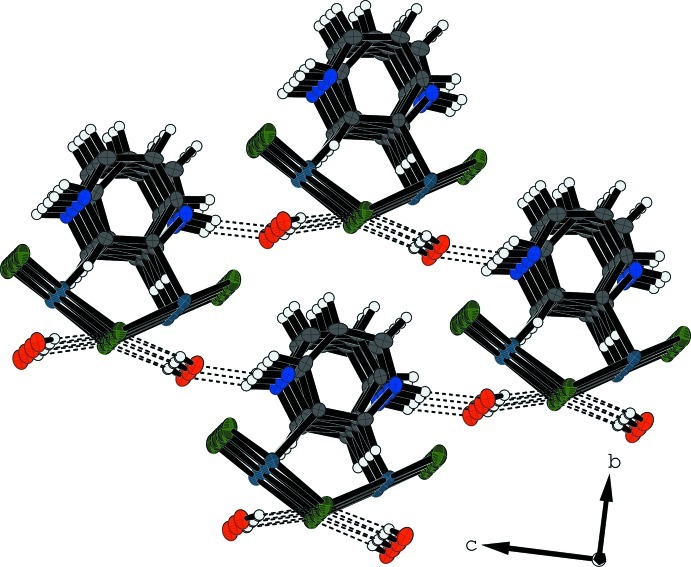
Four neighbouring chains and their hydrogen bond interactions. Thermal displacement ellipsoids are drawn at the 50% probability level. Hydrogen bonds are indicated by dashed lines. Colour code: H white, C grey, N blue, O red, Cl green, As teal.

## supplementary crystallographic information

### Comment

In the course of our work on arylarsonate functionalized metal oxide clusters (see Breen, Clérac *et al. (*2012), Breen, Zhang *et al.* (2012) and references therein for vanadium oxide clusters, Onet *et al.* (2011) for molybdenum oxide clusters and Zhang *et al.* (2012) for manganese oxide clusters) we prepared the title compound, whose crystal structure had until now not been determined. In general, the structural chemistry of organohaloarsenates(III) has not been the object of intense study, the only relevant peer-reviewed publication known to us being the contribution by Grewe *et al.* (1998), which is summarized below. The homologous organohalobismuthates(III) and organohaloantimonates(III) have been more widely studied.

Organodichloroarsines compounds are known to act as soft Lewis acids and form adducts with chloride anions, *e.g.* [RAsCl_3_]^-^ (*R* = Me, Et, Ph) and [MeAsCl_2_(µ-Cl)AsCl_2_Me]^-^. In these adducts, arsenic occurs in ψ-trigonal bipyramidal fourfold coordination. Pentacoordinate, ψ-octahedral adducts are only known for the bromo analogues on account of their stronger acidity: organotribromoarsenates(III) form doubly bridged dimers [RBr_2_As(µ-Br_2_)AsBr_2_R]^2-^ (*R* = Me, Et, Ph) (Grewe *et al.*, 1998). The homologous organohaloantimonates(III) and organohalobismuthates(III) form a wider variety of structures based mostly on ψ-octahedral RE*X*_4_ motifs. These units are found isolated in [PhSbCl_4_]^2-^ (Hall & Sowerby, 1988), [PhSbBr_4_]^2-^ (James *et al.*, 1999) and [MeSbI_4_]^2-^ (Breunig *et al.*, 1992). The ψ-trigonal bipyramidal [PhSbCl_3_]^-^ does exist (Hall & Sowerby, 1988), but its ψ-octahedral dimer [PhCl_2_Sb(µ-Cl)_2_SbCl_2_Ph]^2-^ is also attested (Preut *et al.*, 1987). [Me_2_Sb_2_Cl_6_]^2-^ (Breunig *et al.*, 2010), [Me_2_Sb_2_Br_6_]^2-^ (Althaus *et al.*, 1999), [Ph_2_Sb_2_I_6_]^2-^ (Breunig *et al.*, 1999) and [Ph_2_Bi_2_Br_6_]^2-^ (James *et al.*, 1999) form analogous structures. Addition of a further halide ion results in structures of the [*RX*_3_E(µ-*X*)E*X*_3_R]^3-^ type such as [Ph_2_Sb_2_Cl_7_]^3-^ and [Ph_2_Sb_2_Br_7_]^3-^ (Sheldrick & Martin, 1992). The highest aggregation is found in [{PhSbI(µ^2^-I)}_4_(µ^4^-I)]^-^ (von Seyerl *et al.*, 1986), which features an I_4_ square with Sb atoms on the edges and another I atom in the centre of the square. In all organohaloarsenate(III), -antimonate(III) and -bismuthate(III) structures, bonds to bridging halide ligands are considerably longer than bonds to terminal halide ligands and bridging halide ligands are coordinated *cis* to one another.

In the title compound, the acidity of the aryldichloroarsine is enhanced by the electron-withdrawing, positively charged pyridinio substituent, so that a ψ-octahedral structure similar to the one encountered in [RBr_2_As(µ-Br_2_)AsBr_2_R]^2-^ or [PhCl_2_Sb(µ-Cl)_2_SbCl_2_Ph]^2-^ becomes possible: As is coordinated axially to the organic substituent and equatorially to four chloride ligands, two bridging and two terminal. The terminal chloride ligands are located *cis* to one another and connected to As by short bonds, while the bridging chloride ligands are much more distant from the As centre. In contrast to in the compounds cited above, these bridging chloride ligands do not link to the same organoarsenic unit, but to two different arsenic atoms, thus forming a polymeric chain running along the crystallographic *a* axis (*cf.* Figure 1).

The nitrogen atom on the pyridine ring forms an N—H···O hydrogen bond to a co-crystallized water molecule. The two O—H bonds of this water molecule in turn form O—H···Cl hydrogen bonds to two bridging chloride ions on a neighboring polymeric chain. Through the intermediary of the intervening water molecules, each chain thus acts as a hydrogen bond donor towards the neighbouring chains in the [011] and [011] directions and as a hydrogen bond acceptor towards the neighbouring chains in the [011] and [011] directions (*cf.* Figure 2). It is the lack of centrosymmetry in the hydrogen bonding pattern that accounts for the non-centrosymmetric crystal structure and space group.

The arsenic atom, the pyridinium ring and the oxygen atom of the water molecule all lie in the same mirror plane (Wyckoff position 2 b). The displacement ellipsoids on the pyridinium ring suggest a slight disorder about this mirror plane, but refining this disorder did not improve the quality of the structural model significantly. The bridging chlorine atom (Cl2) lies on a twofold axis (Wyckoff position 2a).

### Experimental

The synthesis reported by Binz & von Schickh (1936) was slightly modified as follows: 3-aminopyridine (20 mmol, 1.882 g) was dissolved in conc. HCl (32%, 15 ml) and As_2_O_3_ (20 mmol, 3.957 g) and CuCl (2 mmol, 0.198 g) were added to the solution. The solution was cooled in an ice bath to -5 °C. NaNO_2_ (30 mmol, 2.07 g) was dissolved in water (3 ml) and slowly added to the solution while keeping the temperature below 0 °C. The reaction mixture was then stirred at 35 °C for 8 h. After cooling to r.t. the precipitate was filtered off and washed with 1-molar HCl solution (2 × 20 ml). The filtrate was cooled to -20 °C and after 7 days the product was obtained in the form of large colourless needles.

### Refinement

H atoms on the pyridine aromatic ring were positioned geometrically and refined using a riding model with C—H distances constrained to 0.95 Å and the N—H distance constrained to 0.88 Å. *U*_iso_ for these hydrogen atoms was constrained to *U*_iso_(H) = 1.2 *U*_eq_(C or N). Restraints were applied to the O1—H1 distance and the H1—O1—H1 angle and *U*_iso_ was constrained to *U*_iso_(H1) = 1.5 *U*_eq_(O1). The anisotropic displacement parameters of the atoms in the aromatic ring indicate a slight disorder around the mirror plane, but refining this disorder did not improve the structural model significantly.

### Crystal data

**Table:**

[AsCl_3_(C_5_H_5_N)]·H_2_O	*D*_x_ = 1.987 Mg m^−^^3^
*M**_r_* = 278.39	Mo *K*α radiation, λ = 0.71073 Å
Orthorhombic, *I**m*2*a*	Cell parameters from 2911 reflections
*a* = 8.2107 (9) Å	θ = 2.8–28.2°
*b* = 8.5812 (9) Å	µ = 4.46 mm^−^^1^
*c* = 13.2046 (14) Å	*T* = 200 K
*V* = 930.37 (17) Å^3^	Block, colourless
*Z* = 4	0.5 × 0.2 × 0.2 mm
*F*(000) = 544	

### Data collection

**Table:**

Bruker SMART APEX CCD diffractometer	1184 independent reflections
Radiation source: fine-focus sealed tube	1167 reflections with *I* > 2σ(*I*)
Graphite monochromator	*R*_int_ = 0.068
φ and ω scans	θ_max_ = 28.2°, θ_min_ = 2.8°
Absorption correction: multi-scan (*SADABS*; Bruker, 1997)	*h* = −10→10
*T*_min_ = 0.341, *T*_max_ = 0.469	*k* = −11→9
3423 measured reflections	*l* = −13→17

### Refinement

**Table:**

Refinement on *F*^2^	Secondary atom site location: difference Fourier map
Least-squares matrix: full	Hydrogen site location: inferred from neighbouring sites
*R*[*F*^2^ > 2σ(*F*^2^)] = 0.032	H atoms treated by a mixture of independent and constrained refinement
*wR*(*F*^2^) = 0.078	*w* = 1/[σ^2^(*F*_o_^2^) + (0.0554*P*)^2^] where *P* = (*F*_o_^2^ + 2*F*_c_^2^)/3
*S* = 1.10	(Δ/σ)_max_ < 0.001
1184 reflections	Δρ_max_ = 1.14 e Å^−^^3^
66 parameters	Δρ_min_ = −1.28 e Å^−^^3^
3 restraints	Absolute structure: Flack (1983), 529 Friedel pairs
Primary atom site location: structure-invariant direct methods	Flack parameter: 0.006 (12)

### Special details

**Table:**

Experimental. R(int) was 0.0767 before and 0.0385 after correction. The Ratio of minimum to maximum transmission is 0.7260. The λ/2 correction factor is 0.0015.
Geometry. All e.s.d.'s (except the e.s.d. in the dihedral angle between two l.s. planes) are estimated using the full covariance matrix. The cell e.s.d.'s are taken into account individually in the estimation of e.s.d.'s in distances, angles and torsion angles; correlations between e.s.d.'s in cell parameters are only used when they are defined by crystal symmetry. An approximate (isotropic) treatment of cell e.s.d.'s is used for estimating e.s.d.'s involving l.s. planes.
Refinement. Refinement of *F*^2^ against ALL reflections. The weighted *R*-factor *wR* and goodness of fit *S* are based on *F*^2^, conventional *R*-factors *R* are based on *F*, with *F* set to zero for negative *F*^2^. The threshold expression of *F*^2^ > σ(*F*^2^) is used only for calculating *R*-factors(gt) *etc*. and is not relevant to the choice of reflections for refinement. *R*-factors based on *F*^2^ are statistically about twice as large as those based on *F*, and *R*- factors based on ALL data will be even larger.

### Fractional atomic coordinates and isotropic or equivalent isotropic displacement parameters (Å^2^)

**Table:**

	*x*	*y*	*z*	*U*_iso_*/*U*_eq_	
As1	0.7500	0.27357 (4)	0.63175 (3)	0.01702 (14)	
Cl1	0.55187 (9)	0.36810 (11)	0.73375 (6)	0.0270 (2)	
Cl2	0.5000	0.15046 (13)	0.5000	0.0229 (2)	
N3	0.7500	0.5797 (5)	0.3838 (3)	0.0215 (8)	
H3	0.7500	0.5690	0.3175	0.026*	
C1	0.7500	0.4632 (5)	0.5450 (3)	0.0187 (8)	
C2	0.7500	0.4508 (5)	0.4411 (3)	0.0201 (8)	
H2	0.7500	0.3509	0.4100	0.024*	
C4	0.7500	0.7237 (6)	0.4225 (4)	0.0291 (11)	
H4	0.7500	0.8117	0.3788	0.035*	
C5	0.7500	0.7435 (6)	0.5249 (4)	0.0402 (14)	
H5	0.7500	0.8450	0.5536	0.048*	
C6	0.7500	0.6127 (6)	0.5865 (4)	0.0333 (12)	
H6	0.7500	0.6250	0.6580	0.040*	
O1	0.7500	0.0724 (4)	0.3215 (2)	0.0242 (7)	
H1	0.672 (2)	0.093 (6)	0.358 (2)	0.036*	

### Atomic displacement parameters (Å^2^)

**Table:**

	*U*^11^	*U*^22^	*U*^33^	*U*^12^	*U*^13^	*U*^23^
As1	0.0145 (2)	0.0197 (2)	0.0169 (2)	0.000	0.000	0.00251 (18)
Cl1	0.0210 (3)	0.0386 (5)	0.0213 (4)	0.0061 (3)	0.0042 (3)	0.0002 (3)
Cl2	0.0192 (5)	0.0278 (6)	0.0219 (5)	0.000	0.0024 (4)	0.000
N3	0.0224 (19)	0.025 (2)	0.0172 (15)	0.000	0.000	0.0015 (14)
C1	0.0204 (19)	0.018 (2)	0.0173 (19)	0.000	0.000	−0.0001 (16)
C2	0.0194 (19)	0.017 (2)	0.024 (2)	0.000	0.000	−0.0017 (16)
C4	0.034 (3)	0.021 (2)	0.032 (2)	0.000	0.000	0.0019 (18)
C5	0.072 (4)	0.014 (3)	0.034 (3)	0.000	0.000	−0.003 (2)
C6	0.058 (4)	0.025 (3)	0.017 (2)	0.000	0.000	−0.0035 (18)
O1	0.0180 (15)	0.0329 (19)	0.0218 (15)	0.000	0.000	−0.0053 (14)

### Geometric parameters (Å, º)

**Table:**

As1—C1	1.990 (4)	C2—H2	0.9500
As1—Cl1	2.2624 (8)	C4—C5	1.363 (7)
As1—Cl2	2.8907 (5)	C4—H4	0.9500
N3—C4	1.337 (7)	C5—C6	1.386 (7)
N3—C2	1.340 (6)	C5—H5	0.9500
N3—H3	0.8800	C6—H6	0.9500
C1—C2	1.377 (6)	O1—H1	0.818 (17)
C1—C6	1.395 (7)		
			
C1—As1—Cl1	92.83 (9)	N3—C2—C1	119.9 (4)
C1—As1—Cl2	87.28 (9)	N3—C2—H2	120.0
Cl1—As1—Cl1^i^	91.95 (4)	C1—C2—H2	120.0
Cl1—As1—Cl2^ii^	179.25 (3)	N3—C4—C5	119.6 (5)
Cl1—As1—Cl2	88.78 (2)	N3—C4—H4	120.2
Cl2^ii^—As1—Cl2	90.49 (2)	C5—C4—H4	120.2
As1^iii^—Cl2—As1	137.13 (5)	C4—C5—C6	118.7 (4)
C4—N3—C2	123.2 (4)	C4—C5—H5	120.6
C4—N3—H3	118.4	C6—C5—H5	120.6
C2—N3—H3	118.4	C5—C6—C1	121.0 (4)
C2—C1—C6	117.5 (4)	C5—C6—H6	119.5
C2—C1—As1	120.7 (3)	C1—C6—H6	119.5
C6—C1—As1	121.8 (3)		
			
C1—As1—Cl2—As1^iii^	−26.08 (9)	C6—C1—C2—N3	0.000 (2)
Cl1—As1—Cl2—As1^iii^	66.82 (2)	As1—C1—C2—N3	180.000 (1)
Cl2^ii^—As1—Cl2—As1^iii^	−113.33 (2)	C2—N3—C4—C5	0.000 (2)
Cl1—As1—C1—C2	−133.95 (2)	N3—C4—C5—C6	0.000 (2)
Cl2—As1—C1—C2	−45.308 (11)	C4—C5—C6—C1	0.000 (2)
Cl1—As1—C1—C6	46.05 (2)	C2—C1—C6—C5	0.000 (2)
Cl2—As1—C1—C6	134.692 (12)	As1—C1—C6—C5	180.000 (2)
C4—N3—C2—C1	0.000 (2)		

Symmetry codes: (i) −*x*+3/2, *y*, *z*; (ii) *x*+1/2, *y*, −*z*+1; (iii) −*x*+1, *y*, −*z*+1.

### Hydrogen-bond geometry (Å, º)

**Table:**

*D*—H···*A*	*D*—H	H···*A*	*D*···*A*	*D*—H···*A*
O1—H1···Cl2	0.82 (2)	2.40 (2)	3.197 (2)	165 (3)
N3—H3···O1^iv^	0.88	1.84	2.711	173

Symmetry code: (iv) −*x*+3/2, *y*+1/2, −*z*+1/2.

## References

[bb1] Althaus, H., Breunig, H. J. & Lork, E. (1999). *Chem. Commun.* pp. 1971–1972.

[bb2] Binz, A. & von Schickh, O. (1936). German Patent DE 633867.

[bb3] Breen, J. M., Clérac, R., Zhang, L., Cloonan, S. M., Kennedy, E., Feeney, M., McCabe, T., Willams, D. C. & Schmitt, W. (2012). *Dalton Trans.* **41**, 2918–2926. 10.1039/c2dt11153e 22266646

[bb4] Breen, J. M., Zhang, L., Clement, R. & Schmitt, W. (2012). *Inorg. Chem.* **51**, 19–21. 10.1021/ic202104z 22145924

[bb5] Breunig, H. J., Denker, M. & Lork, E. (1999). *Z. Anorg. Allg. Chem.* **625**, 117–120.

[bb6] Breunig, H. J., Ebert, K. H., Gülec, S., Dräger, M., Sowerby, D. B., Begley, M. J. & Behrens, U. (1992). *J. Organomet. Chem.* **427**, 39–48.

[bb7] Breunig, H. J., Koehne, T., Lork, E., Moldovan, O., Poveleit, J. & Raţ, C. I. (2010). *Z. Naturforsch. Teil B*, **65**, 1245–1248.

[bb8] Bruker (1997). *SMART, *SAINT* and *SADABS** Bruker AXS Inc., Madison, Wisconsin, USA.

[bb9] Dolomanov, O. V., Bourhis, L. J., Gildea, R. J., Howard, J. A. K. & Puschmann, H. (2009). *J. Appl. Cryst.* **42**, 339–341.

[bb10] Flack, H. D. (1983). *Acta Cryst.* A**39**, 876–881.

[bb11] Grewe, S., Häusler, T., Mannel, M., Rossenbeck, B. & Sheldrick, W. S. (1998). *Z. Anorg. Allg. Chem.* **624**, 613–619.

[bb12] Hall, M. & Sowerby, D. B. (1988). *J. Organomet. Chem.* **347**, 59–70.

[bb13] James, S. C., Norman, N. C. & Orpen, A. G. (1999). *J. Chem. Soc. Dalton Trans.* pp. 2837–2843.

[bb14] Onet, C. I., Zhang, L., Clérac, R., Jean-Denis, J. B., Feeney, M., McCabe, T. & Schmitt, W. (2011). *Inorg. Chem.* **50**, 604–613. 10.1021/ic101672t 21141887

[bb15] Preut, H., Huber, F. & Alonzo, G. (1987). *Acta Cryst.* C**43**, 46–48.

[bb16] Seyerl, J. von, Scheidsteger, O., Berke, H. & Huttner, G. (1986). *J. Organomet. Chem.* **311**, 85–89.

[bb17] Sheldrick, G. M. (2008). *Acta Cryst.* A**64**, 112–122. 10.1107/S0108767307043930 18156677

[bb18] Sheldrick, W. S. & Martin, C. (1992). *Z. Naturforsch. Teil B*, **47**, 919–924.

[bb19] Zhang, L., Clérac, R., Heijboer, P. & Schmitt, W. (2012). *Angew. Chem. Int. Ed.* **51**, 3007–3011. 10.1002/anie.201107358 22311623

